# Polysaccharide/Carbon Quantum Dots Composite Film on Model Colloidal Particles—An Electro-Optical Study

**DOI:** 10.3390/polym15183766

**Published:** 2023-09-14

**Authors:** Viktoria Milkova

**Affiliations:** Institute of Physical Chemistry ‘Acad. Rostislaw. Kaischew’, 1113 Sofia, Bulgaria; vmilkova@ipc.bas.bg

**Keywords:** carbon dots, polysaccharides, layer-by-layer assembly, electrokinetic spectroscopy

## Abstract

Negatively charged carbon dots (Cdots) were successfully impregnated into chitosan/alginate film formed on model colloidal particles as a result of the attractive interactions with the chitosan molecules. The electrical properties of the produced films were studied by electrokinetic spectroscopy. In this study, the electric light scattering method was applied for first the time for the investigation of suspensions of carbon-based structures. The electro-optical behavior for the suspension of polymer-coated particles showed that the electric polarizability of the particle-covered layer from alginate was significantly higher compared to that of the layer from chitosan due to the higher charge density of alginate. The presence of a low concentration of Cdots in the film results in partial charge screening. It was confirmed that the polarizability of counterions with lower mobility along the adsorbed polyion chains was responsible for the registered electro-optical effect from the suspension of polymer-coated particles and that the participation of diffuse H^+^ counterions of Cdots in the creation of the electro-optical effect was negligible. The observed oscillation behavior in the evolution of the film thickness was interpreted through the participation of compensatory effects due to the additional adsorption/desorption of polyelectrolyte complexes from the film surface. The concentration of Cdots in the film was determined, and the loaded amount was ca. 6.6 µg/mL per layer.

## 1. Introduction

The self-assembly of nanosized species into larger structures is a promising approach for the development of functional composite platforms, with potential applications in biotechnology, pharmacy, and medicine [1,2,3]. Among the various procedures for developing new biocompatible structures, the systems based on a sequential electrostatic deposition of oppositely charged components on charged surfaces offered a possibility for the successful inclusion of different bioactive molecules. In essence, the layer-by-layer method is a sequential electrostatic assembly process, where the charged substrate is alternately exposed to contact with solutions of two oppositely charged polyelectrolytes and finally results in a polyelectrolyte complex, stabilized by strong electrostatic forces [4]. The procedure allows the incorporation of various small, charged components into the film, resulting in a multi-component shell with specific properties [5]. Along these lines, impregnating carbon-based nanomaterials (carbon dots, onions, nanotubes) into self-assembled polymer films is a promising procedure for the development of functional core–shell structures [6,7]. 

Cdots are quasi-spherical structures (up to 10 nm) [8,9]. They possess distinctive advantages over classical heavy metal-based semiconductor quantum dots (Qdots)—high solubility and stability in water, bright photoluminescence in the visible spectrum, low cytotoxicity, biocompatibility, high chemical stability and photostability, easy functionalization, and low-cost synthesis [10,11,12]. Their remarkable features create the opportunity for a great diversity of applications—biosensing, cell imaging, drug delivery, detection of metal ions, catalysis and photovoltaic or optoelectronic devices, and energy conversion/storage. Cdots possess different surface functional groups (amino, hydroxyl, carboxylic, ether, and epoxy) that ensure their high hydrophilicity and their possibility for functionalization with organic, polymeric, or inorganic species [13]. Therefore, the impregnation of Cdots in polyelectrolyte microcapsules produced via the subsequent deposition of oppositely charged polymers holds promise as an effective approach for the design of structures with competitive potential for applications in biotechnology for imaging, drug delivery, and biosensors [14,15,16]. Moreover, because of the versatility of the properties and dimensions, Cdots have promising potential for the design of functional coverages [17]. 

The present study aims to investigate polysaccharide/Cdot multilayers formed on model particles. The electro-optical method of electric light scattering was applied for the first time to study the electrical and geometrical properties of carbon-based structures. The electro-optical phenomena are manifestations of the changes in the optical behavior of the dispersion of anisometric colloid particles upon the applied electric field. The method makes it possible to obtain information about the electrical, geometrical, and optical properties of the particles. The method is very sensitive and can give information about the properties of non-spherical colloidal particles in the presence of low molecular electrolytes, surfactants, or polymers. The core of the model structures were β-FeOOH particles. These oxide particles were used for the electro-optical investigation because of their ellipsoidal shape and narrow size distribution. Moreover, the electro-optical behavior and the electrokinetic properties of bare β-FeOOH particles in suspension or particles with adsorbed polymers are very well studied and have been reported by Radeva and co-authors [18,19,20]. 

The Cdots were impregnated into a multilayer formed from chitosan and alginate. Chitosan is obtained by deacetylation of its parent polymer chitin, a polysaccharide widely distributed in nature (in the exoskeletons of insects and crustaceans and in certain fungi) [18]. The polymer is biocompatible and non-toxic to living tissues, and it has antibacterial, antifungal, and antitumor activity. That is why it has various biomedical and pharmaceutical applications, such as wound healing, drug delivery, gene therapy, tissue engineering, and bioimaging [21]. 

Alginate is a natural polysaccharide produced by brown seaweeds and marine algae. It is a water-soluble linear block copolymer. The molecular structure can be described as a block-wise pattern of homo-polymeric regions of (1→4)-linked poly–L– α guluronate residues (G-block) and homo-polymeric regions of poly–D– β mannuronate residues (M-block) interspersed by MG-blocks [22,23]. The presence of carboxylic groups along the molecule governs the polyanionic behavior of the molecule. Alginate meets all the requirements for its use in pharmaceutical and medical applications—it is a biodegradable, biocompatible, and mucoadhesive polymer [24,25,26,27,28,29].

The investigation was performed on the basis of the working hypothesis that stable polysaccharide-based structures can be formed in spite of the low charge density and concentration of Cdots and that their presence reflects the electrical properties of the composite film. This finding is in line with our previous studies on the electrical properties of polyelectrolyte multilayers on colloids. The analysis of the electrical properties of the particles covered with film took place within the framework of the classical counterion condensation theory [30]. The theory describes the interaction of monovalent counterions with polyions with a high charge density. In the present experimental conditions, chitosan and alginate are fully charged polyelectrolytes. Therefore, the presence of counterions with different mobility near the polyion vicinity can be expected. The application of electro-optics is a useful tool for distinguishing the participation of these ions in the electrical properties of the particles covered with polymer film. 

## 2. Materials and Methods

### 2.1. Materials

The multilayer film was formed on particles synthesized following a well-established experimental procedure [31].

Chitosan (CS) (degree of deacetylation, DA 75–85%, molecular weight, Mw 50–190 kDa) and alginate, sodium salt (ALG) (low viscosity, Mw 538 kDa) were obtained from Sigma–Aldrich (Sigma Aldrich, Taufkirchen, Germany). The stock solutions of polysaccharides were prepared with a concentration of 1 mg/mL in a hydrochloric solution (5% stoichiometric excess of HCl, for chitosan) or double-distilled water (for alginate). Cdots, CDs (dispersion, particle diameter ca. 10 nm) were purchased from Merck (Merck, München, Germany).

### 2.2. Methods

#### 2.2.1. Electric Light Scattering

The electro-optical phenomena in colloidal systems are manifestations of the changes in their optical behavior upon the applied electric field. It can allow the obtaining of information about the electrical, geometrical, and optical properties of the anisometric particles in the system arising from the mobility and distribution of the particle electrical charges. 

The orientation of particles by an externally applied electric field results from the interaction between its electric moments (permanent and induced) and the orienting field. As a consequence of the orientation, the light scattered by the suspension is changed. The electro-optical effect, α, is defined as the difference between the intensities of the scattered light from the suspension in the presence and absence of an electric field [32]:

α = (I_E_ − Io) × 100/Io (1)

The method is very sensitive when used for the characterization of the electrical and geometrical properties of particles with sizes up to 1 μm in a suspension with a low ionic strength (thick electric double layer, EDL) if the particle’s refraction coefficient is different from that of the water. In order to prevent multi-scattering from the suspension, the concentration of the particles must be low. In this experiment, the electric light scattering is recorded at an angle of 90° with respect to the electric field, using white unpolarized light. 

Generally, in the electro-optical experiment, when the sinusoidal electric field is applied to the colloid system, two electro-optical effects are observed. The first one appears in the range of the particle rotation (at low frequencies, below 1 kHz) and seems to be related to its permanent dipole moment. The second effect (at kilohertz frequencies) is related to the polarization of ions in the diffuse part (free ions) of the electrical double layer (EDL) of the particle. In a suspension stabilized by the adsorption of a fully charged polyelectrolyte, an additional effect appears near the range of the particle rotation. It is attributed to the polarization of a layer from the condensed counterions (CC) near the polyion surface. It is suggested that the mobility of the CC is lower than the free ion’s mobility because of the strong attraction to the polyion surface. 

When an electric field with ν = 0 Hz is applied to a suspension of non-spherical particles, all the charges of the particles are polarized (ions in the Stern layer, ions from the diffuse part of the EDL, and associated ions). When a sinusoidal electric field (ν > 0 Hz) is applied, the orientation of the particles follows the field. At low frequencies of the field (ν < 1 kHz) the particle rotation is observed. At higher frequencies (or by decreasing the period of the field), the particles cannot follow the field and the plateau region in the frequency dependency is achieved. The registered effect results from the polarization of the free ions from the diffuse part of the EDL of the particles. 

According to the electro-optical theory, at a low energy of orientation, where the energy of the orientation of the particles is lower than kT, a linear dependence on the square of the electric field strength, E^2^, is expected. For a stable suspension of particles that do not have a permanent dipole moment, the initial slope of the dependence directly reflects changes in the particle’s electrical polarizability, γ [33]:

α = A(Ka, Kb) (p’^2^/kT + (γ_a_ − γ_b_)E^2^/4 kT)/I_0_(Ka, Kb) (2)

where A(Ka, Kb) and I_0_(Ka, Kb) are optical functions that depend on the particles sizes and on K = 2π/λ sin(θ/2) (where λ = 547 nm is the wavelength of the incident light and θ is the angle of observation); p’, γ_a_, and γ_b_ are the permanent dipole moment and the electrical polarizability with respect to the long and transverse axes of the particle. 

#### 2.2.2. Potentiometric Acid-Base Titration

The surface charge density of β-FeOOH particles was measured by acid–base potentiometric titration. In the experimental procedure, the addition of a volume of titrant and the measurements were computer controlled. In order to obtain the dependence of the surface charged density as a function of the pH of the particles, a suspension (30 mL) containing particles with a concentration 8 µg/mL in the presence of 10^−4^ M NaCl was titrated. The charge density of the particles at a given pH value was calculated as the difference between the amount of H+ or OH- ions added to the suspension and the amount added to a blank solution (solvent) with the equivalent volume and electrolyte concentration as the colloid suspension. The amount of titrant used to produce a given pH value in the suspension was then diminished with the amount necessary to produce this given pH value in the blank solution. The difference then constituted the proton consummation at a given pH and corresponded to the surface charge density of the particles. 

#### 2.2.3. Microelectrophoresis

The electrophoretic mobility, U_ef_, of the particles after each deposition was measured using a Rank Brothers Mark II (Rank Brothers, Newbury, UK) apparatus with a flat quartz cell at 25 °C. The calculation of U_ef_ was conducted as described in a previous study [34]. 

#### 2.2.4. Dynamic Light Scattering

The comparative measurements of the surface charge and hydrodynamic diameter of the bare oxide particle and Cdots were performed using dynamic light scattering with non-invasive backscattering (DLS-NIBS, measuring angle 173°). The measurements were carried out using Zatasizer Pro (Malvern Panalytical Ltd., Malvern, UK) equipped with a He-Ne laser with a maximum power of 10 mW, operating at a wavelength of 633 nm with a fixed scattering light angle of 173°. All the measurements were performed at 24.0 ± 0.1° 

#### 2.2.5. Formation of the Composite Multilayer Film

**Figure 1:**
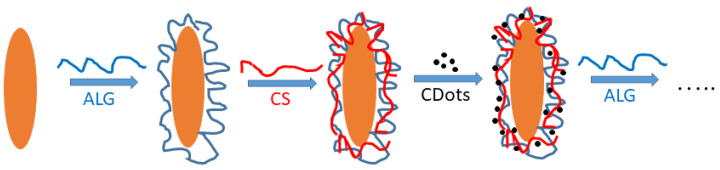
Deposition steps in the experimental procedure for preparation of alginate/chitosan/Cdots film on β-FeOOH particles.

The film was formed by consistent adsorption of oppositely charged alginate, chitosan, and Cdots on the positively charged core (Figure 1). Briefly, the suspension of bare particles was prepared with a concentration (8 µg/mL) in a solution of HCl (10^−4^ M, pH 4.02). Then, the first polymer layer was formed by adding 19.8 mL from a dispersion of positively charged particles to the solution of negatively charged alginate (0.2 mL from the stock polymer solution, final concentration 100 µg/mL) and stirring for 20 min. This procedure was repeated by adding the ALG-coated particles to the solution of oppositely charged chitosan (100 µg/mL). The negatively charged Cdots (final concentration 10 µg/mL) were impregnated after each chitosan layer by using the same procedure. The free polysaccharide molecules or Cdots were not removed from the suspension after the deposition steps. For the formation of each subsequent layer, 19.8 mL from the suspension of particles with a previously adsorbed polymer layer and 0.2 mL of the stock solution of the polymer were added to the next layer. This procedure prevented the increase in the volume of the dispersion during the formation of the film.

The absence of centrifugation after the polymer deposition enabled the control of the amount of each component in the dispersion during the process of film formation; this was useful for the determination of the amount of Cdots loaded into the film. The pH of ca. 4 and the ionic strength of the dispersion were kept almost constant during the deposition process. 

#### 2.2.6. An Estimation of the Hydrodynamic Thickness of Each Polymer Layer

The dimensions of the particles were estimated using the electro-optical method. The rotation diffusion coefficient, D_r_, relative to the dimensions of the particles, was calculated from the relaxation time, τ, of the Brownian disorientation after switching the applied electric field off:

D_r_ = 1/6τ (3)

For the prolate ellipsoid, D_r_ is given by Perrin’s equations:

D_r_ = kTp^2^ [[(−1 + (2p^2^ − 1)/2pB)] × ln[(p + B)/(p − B)]]/4ηπ(p^4^ − 1) (4)

B^2^ = p^2^ − 1 (5)

where p is the axial ratio a/b, η is the viscosity of the suspending medium, k is the Boltzmann constant, T is the temperature, and υ is the volume of the particle [35].

In order to compare the particle dimensions using DLS, the calculation of the translation diffusion coefficient D_t_ for the prolate ellipsoid is given by

D_t_ = kT G(ρ)/3πηa (6)

G(ρ) = ln(1 + A/ρ)/A(7)

A^2^ = 1 − ρ^2^ (8)

where G(ρ) is the “shape” factor (ρ = 1/p, for prolate ellipsoid ρ < 1).

The hydrodynamic thickness, L_H_, of each polymer layer was calculated from the difference in the size of the particles before and after the deposition step (p decreases with the number of the adsorbed steps):

p = (a + 2L_H_)/(b + 2L_H_) (9)

#### 2.2.7. UV-Vis Spectroscopy

The amount of Cdots loaded into the polysaccharide film was determined using the T60 UV/VIS spectrophotometer (PG Instruments Ltd., Leicestershire, UK). For this purpose, after each deposition of Cdots into the film, aliquots from the suspension were centrifuged (at 4500 rpm, 3147 g for 45 min), and the supernatant was extracted. The concentration of Cdots in the film was determined by the difference between the initial concentration of the compound added to the suspension and the concentration in the supernatant. 

## 3. Results

### 3.1. Surface Charge Density of the Model Oxide β-FeOOH Particles

We used the oxide particles as a model core for the formation of a polyelectrolyte multilayer in some studies [36,37,38]. The electro-optical behavior and the dynamics of the counterion charges in the diffuse part of the double electric layer of the particles in the suspension of bare particles and the particles with an adsorbed polymer layer were studied in detail. In these previous studies, it was assumed that the value of the surface charge density of the bare particles at pH 5 (ca. 0.3 e/nm^2^ or 48 mC/m^2^) was close to one reported in the literature for particles produced using different procedures [39]. That is why the first part of this study was focused on the estimation of the surface charge density of the used oxide particles. 

**Figure 2:**
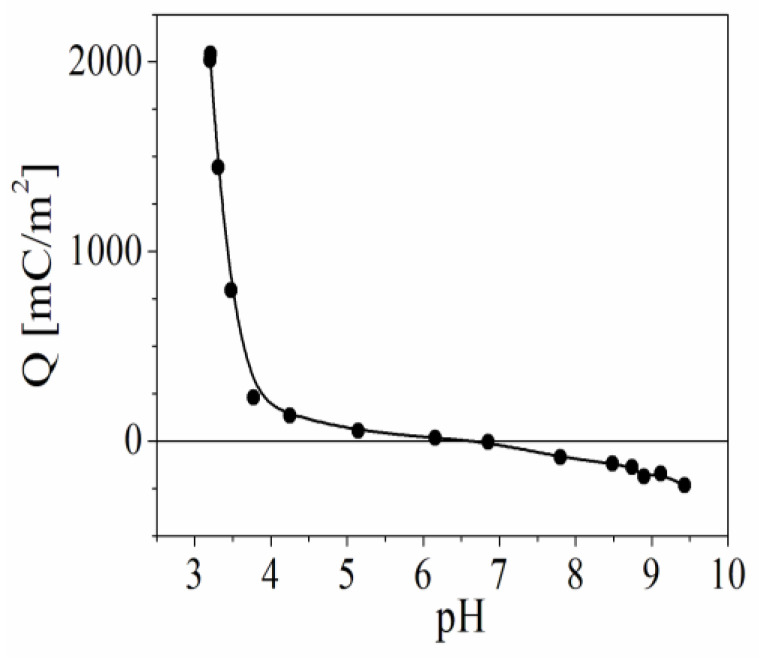
Dependence of the surface charge density of bare β-FeOOH particles as a function of pH of the suspension (in the presence of 10^−4^ M NaCl).

The bare β-FeOOH particles have a constant surface potential, and their surface charge depends on the concentration of H^+^ in the solution. In an acidic medium, the particles are positively charged due to the interaction of protons with hydroxyl groups on the particle surface: 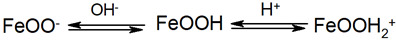


The surface charge of the particles is a very important parameter in multilayer formation, and it was estimated using acid–base potentiometric titration. Figure 2 presents the dependence of the surface charge of the β-FeOOH particles as a function of the pH of the suspension in the presence of 10^−4^ M NaCl. The results show that the point of zero charge (pH_PZC_) is ca. 6.85, which is close to that of the experimental results obtained by Kanungo et al. [39] for particles produced using a different procedure (pH_PZC_ ~7.15). 

In the present experimental conditions (pH 4.7), the particles have a very low surface charge density (17 mC/nm^2^). However, our previous results show that it is high enough to ensure the formation of stable multilayers from polyelectrolytes and hybrid films containing polyelectrolytes and smaller nanoparticles [40]. The charge of the particles strongly increases at the low pH of the solution. 

The nanocrystals from β-FeOOH have a hollandite structure with tetragonal cells and small pores (5 × 5 Å^2^), which contain no stoichiometry ratio of Cl^−^ and water. The presence of specific adsorbed chloride ions is very important for the formation and stability of the produced particles [41]. It is not possible to completely remove the concentration of chloride ions from the fresh suspension (the remainder is ca. 2%). In the presence of a high concentration of a base, the chloride ions can be released from the pores and a change in the particle’s morphology occurs (β-FeOOH particles will transform in α-FeOOH). Therefore, the information about the charge density of the particles at high pH might not correspond completely with the β-FeOOH particles. 

**Figure 3:**
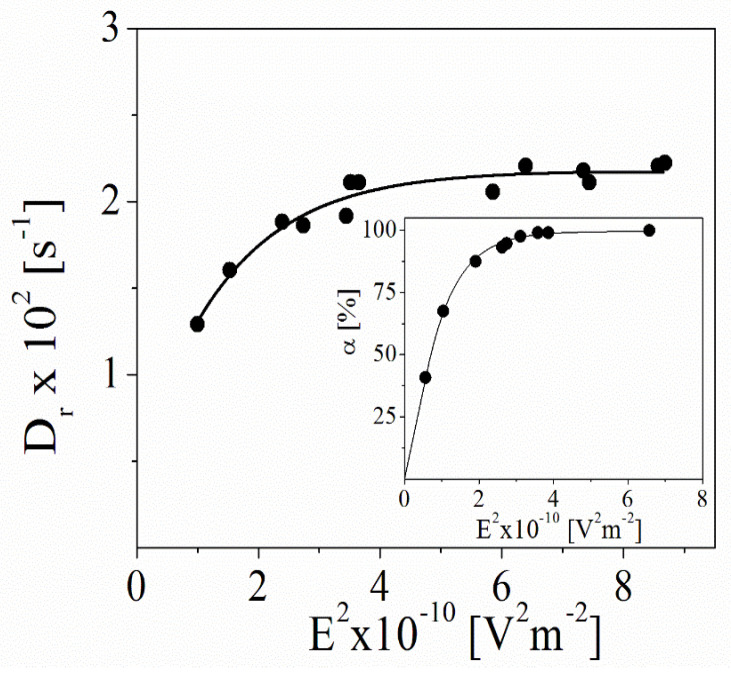
Dependence of the rotary diffusion coefficient of bare β-FeOOH particles as a function of the electric field strength. Inset: dependence of the electro-optical effect as a function of the electric field strength (the frequency of the electric field is 3 kHz).

The electro-optical method was applied to estimate the size of the bare β-FeOOH. Figure 3 shows the dependence of the rotary diffusion coefficient, D_r_, of the particles as a function of the electric field strength. The diffusion coefficient is calculated from the relaxation time of the Brownian disorientation after switching the applied electric field off (D_r_ = 1/6τ). For the prolate ellipsoids, the relation with the particle size is given by the Perrin equation (Equations (3) and (4)).

The produced particles have a very narrow size distribution. However, the dependence shows the presence of bigger particles or aggregates which also participate in the creation of the electro-optical effect of the suspension at low electric fields (or at a low energy of orientation). The results indicate that the D_r_ values are almost constant at a high intensity of the applied electric field, where the achieved orientation of the particles is very close to the full orientation in the system (Inset of Figure 3). 

**Figure 4:**
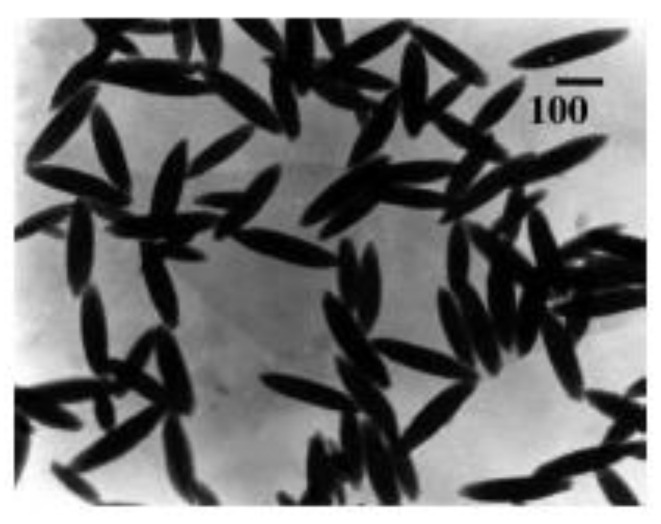
Representative TEM images of β-FeOOH particles. The length dimension bar is 100 nm.

According to the electro-optical theory, at a high electric field when the full orientation of the particles is achieved, the electro-optical effect predominantly depends on the properties of single particles, and the registered relaxation time corresponds to the rotary diffusion coefficient (the size, respectively) of a single particle [32]. The size of the bare β-FeOOH is determined using Perrin’s equation (Equations (4) and (5)), and the particle dimensions (a~300 and b~75 nm for the long and transversal axes; the axial ratio p is 4) are slightly different from those obtained from the electron microscopy (a~285 and b~72 nm; p is 3.96; see Figure 4). 

The comparative analysis of the hydrodynamic size of the particles was performed using the data for the translation diffusion coefficient obtained from DLS (Equations (6) and (8)). The calculated dimensions (a~325 nm, b~81 nm) are very close to the ones estimated by electro-optics.

### 3.2. Characterization of Produced Colloid–Polymer Complexes

**Figure 5:**
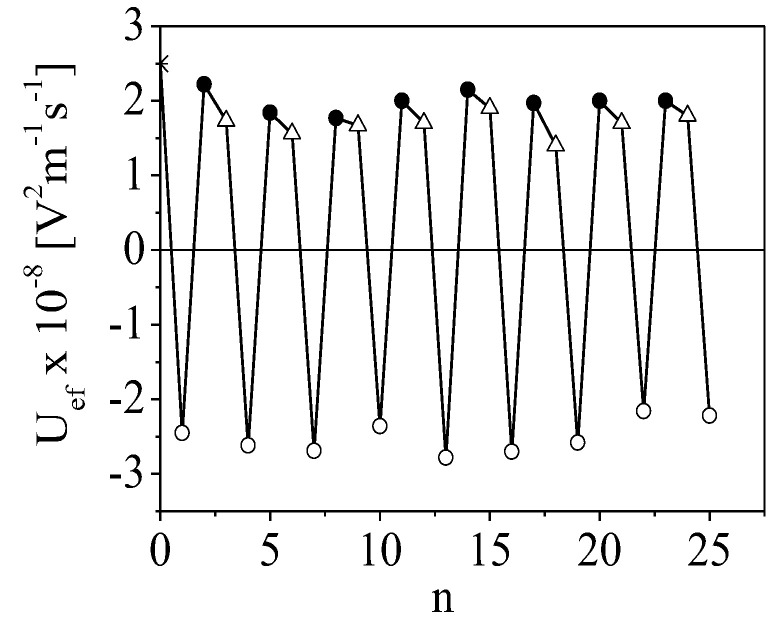
Alteration in the electrophoretic mobility, U_ef_, of β-FOOH particles (∗) covered by film after each adsorption step, n, from ALG (○) and CS (●) layer or Cdots (△).

Figure 5 presents the dependence of the electrophoretic mobility, U_ef,_ of the film-coated particles as a function of the number of adsorption steps. The results indicate an achievement of overcharging after each deposition, which is a key factor for the formation of a stable multilayer. The value of U_ef_ has almost no dependency on the number of steps for each component. However, the absolute values of U_ef_ for the particles with the outer CS layer are slightly lower compared to the particles with the ALG layer due to the lower charge density of chitosan. 

Cdots are impregnated into the film due to the electrostatic interactions with the oppositely charged chitosan molecules adsorbed on the particle surface. The low amount of loaded Cdots results in a partial screening of the charge of chitosan (not overcharging). The sequential adsorption of ALG molecules leads to an overcharging of the particle surface. 

As proof of a successful adsorption process, the electrophoretic measurements can only give information about the overcompensation of the surface charge of the particles and the stability of the dispersion after each deposition step. However, the oscillation in the sign of U_ef_ can also result from the adsorption–desorption processes on the surface. Therefore, more measurements than these are needed for a complete characterization of the electrical properties of the film and the stability of the produced structures. To obtain more detailed information, the electro-optical method was applied. 

**Figure 6:**
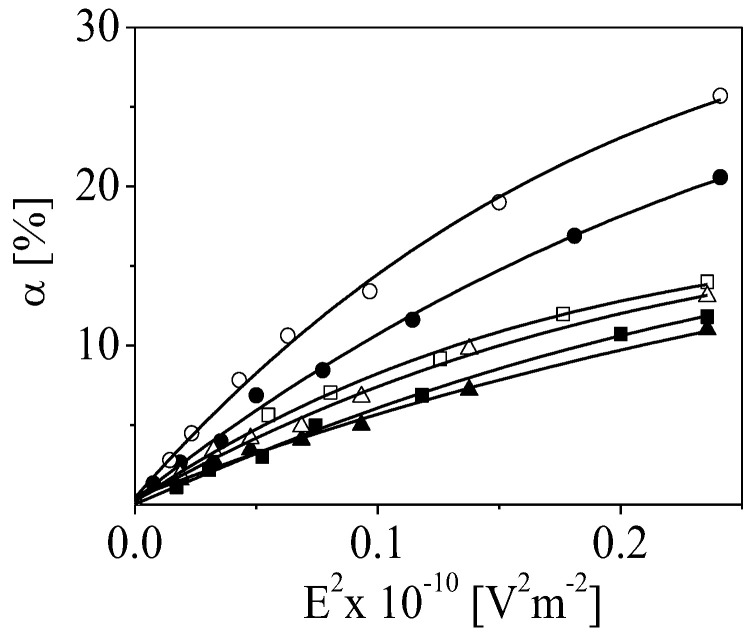
Dependence of the electro-optical effect, α, as a function of the intensity of the applied electric field for particles with the last adsorbed layer from ALG2 (●), ALG8 (○), CS2 (■), CS8 (□), CDs2 (▲), and CDs8 (△). The frequency of the electric field is 1 kHz.

According to the experimental results, the electro-optical effect α increases with the number of adsorbed steps due to the increasing number of adsorbed monomers on the growing particle surface. The electrical polarizabilities of the particles covered with polymer film are calculated from the initial slope of the dependencies presented in Figure 6. 

**Figure 7:**
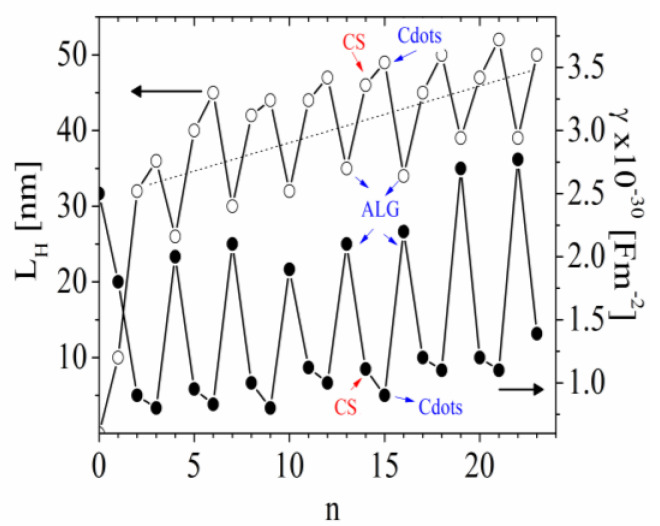
Dependence of the electrical polarizability, γ (●) and hydrodynamic thickness, L_H_ (○) as a function of the number of adsorption steps, n. (The dot line correspond to the line drawn by eye to illustrate the increased thickness of the film with the number of adsorbed layers.)

The estimations indicate that the γ of the suspension of the particles with outer layers from alginate is significantly higher compared to that of the chitosan layers because of the higher charge density of alginate (Figure 7). The deposition of Cdots leads to a slight decrease in the α (or γ with ca. 10%) registered from the suspensions of particles as a result of the partial neutralization of the chitosan charges.

The estimated electrical polarizability γ is an indicator of the surface electrical properties of the particles. The results indicate that the values of γ from a suspension of particles after ALG adsorption correlated with a higher charge density of the polymer. However, the deposition of ALG results in a significant decrease in the film thickness, L_H_ (Figure 7). The correlation between γ and the hydrodynamic thickness of the layer was shown in our previous studies for films formed from CS/pectin and CS/sodium carboxymethyl cellulose [36,37]. In these cases, the oscillation at a higher value of γ is correlated with higher values of L_H_. As the higher polymer charge density increases in the γ of the particles covered with an adsorbed layer, the explanation for the observed correlation with the γ/L_H_ behavior requires analysis of the evolution of the film thickness. 

The thickness of each adsorbed layer is calculated from the difference between the D_r_ of the particles before and after each deposition step (Equation (9)). For the presented system, a few reasons for the observed oscillation in L_H_ are proposed.

First, the oscillation could result from the partial desorption of CS/ALG complexes from the film surface. However, the electro-optical results indicated the achievement of the overcharging of the surface charge and the increasing of the electrical polarizability after each adsorption step.

Second, the lower charge density of CS requires the adsorption of more molecules on the surface in order to ensure surface overcharging [42]. Moreover, we previously showed a similar oscillation for pectin/CS film; in addition, CS forms layers of greater thickness compared to pectin, even when chitosan has a higher charge density [36]. 

Third, the increase in L_H_ after CS adsorption suggested that CS chains might be able to diffuse into the film. The “diffusion” model has been proposed by Lavalle et al. [43] in order to explain the exponential film growth regime of the multilayer. The method is based on the polymer diffusion “in” and “out” of the film formed from oppositely charged polyelectrolytes with different charge densities. 

Radeva et al. [35,36,37,38] have shown exponential or stepwise growth of the multilayer form with chitosan. The authors proposed that the diffusion of CS molecules into the film bulk might occur because of additional ionization of the weak polyanion when the film is brought into contact with a solution of fully charged chitosan. The CS chains form ion pairs with the additionally ionized groups of the polyanion and remain bound during the rinsing step. In new contact with the solution of the polyanion, the overcharging of the surface occurs. Meanwhile, the degree of ionization of the molecules in the previously adsorbed layer decreases and the CS chains diffuse out of the film. These molecules can interact with the polyanion chains coming from the solution and form complexes that can adsorb on the film surface. 

In the present study, the film is formed from fully charged polyelectrolytes. Therefore, if some of the CS chains are able to diffuse “in-out” from the film, these chains do not form additional ion pairs with ALG molecules from the previous layer because of an absence of more groups that are able to dissociate. For that reason, these free chains are able to diffuse out of the film during the rinsing step. However, the CS/ALG film was formed without rinsing after each deposition, and therefore, we assumed that there was a different adsorption mechanism in the build-up process. 

Fourth, the registered higher L_H_ after CS adsorption might be a result of compensation effects. In spite of the low concentration of polymers used for film formation, there are small free polymer molecules in suspension after each step. Some free molecules of ALG can form complexes with CS molecules in the bulk during the second step, and these complexes can deposit on the film surface. The amount of free CS molecules is low, and the formation of complexes in the suspension can be neglected in the third step. A similar mechanism can be applied to the following steps. Hence, it shows that L_H_ increases after CS adsorption. The Cdot adsorption results in a slight increase in L_H_ (ca. 5 nm); then, it significantly decreases after the subsequent ALG deposition. 

Moreover, it was suggested that irregular film growth could also result from the partial desorption of CS molecules from the film when it is exposed to the solution of the longer ALG chains. The combination of polyelectrolytes with strongly different chain lengths is known to favor the formation of quasi-soluble complexes that can be easily re-dissolved [44]. Therefore, in the next deposition step, the equilibrium in the concentration of polysaccharide is disturbed, and the possibility of the formation of complexes in suspension can be expected. 

The proposed compensation effects for the evolution of L_H_ assumed that formation of non-stoichiometry complexation in the film. The charge stoichiometry can be calculated by comparing the electrical polarizability of the particles covered by the ALG or CS layers, γ(ALG)/γ(CS)~2, which indicates the presence of excess negative charges (from alginate) in the bilayer. 

It is well known that the charge into the multilayer is balanced by a combination of oppositely charged monomers and small counterions (“intrinsic” or “extrinsic” charge compensation) [45]. To render the film neutral, the excess charge into the CS/ALG film must be compensated for by an equal excess of hydrated Na+ counterions. Therefore, it is expected a more “soft” hydrated alginate layer will be formed that might influence the electrical properties of the particles when the electric field is applied to the suspension. Previously, we reported the appearance of an unusual manifestation of the electro-optical effect at a high intensity of the electric field registered from suspensions of particles covered by CS/pectin multilayer. According to the reported data for particles with an adsorbed CS layer, at a low energy of orientation, the effect was higher compared to pectin. However, at a high energy of orientation (when the complete orientation of the particles in the suspension is achieved), the experimental curves of the dependences were intersected, and the value of the saturated effect from CS was lower. To explain the observed electro-optical behavior, we assumed that it resulted from an elasto-hydrodynamic effect, which favored the perpendicular orientation of the “soft” particles [38]. A similar effect decrease has been registered for a very thick multilayer, but it has not been observed for a thin film formed from the same polymers in different adsorption conditions [37]. That is why in the present study, it was suggested that there was a correlation between L_H_ (the geometry or an axial ratio of the coated particle) and the elasto-hydrodynamic properties of the film. According to this suggestion, despite the high excess charges in the film, the influence of an elasto-hydrodynamic effect on the electro-optical behavior was not observed. 

### 3.3. Dynamics of the Counterions

The influence of the charge density of the components in the film on the electrical properties of the particles can be distinguished through an analysis of the electro-optical behavior at the low electric field strength. The comparison between the experimental results for the particles with an outer layer from ALG and those of CS indicates that the registered effect is higher but that the relaxation frequency, υ_cr_, of the effect is lower for the film terminated with ALG. (The experimental relaxation frequency of the kilohertz electro-optical effect, defined as a frequency for a two-fold decrease in the effect value, υ_cr_, is related to the mobility of the counterions responsible for creating the effect.)

**Figure 8:**
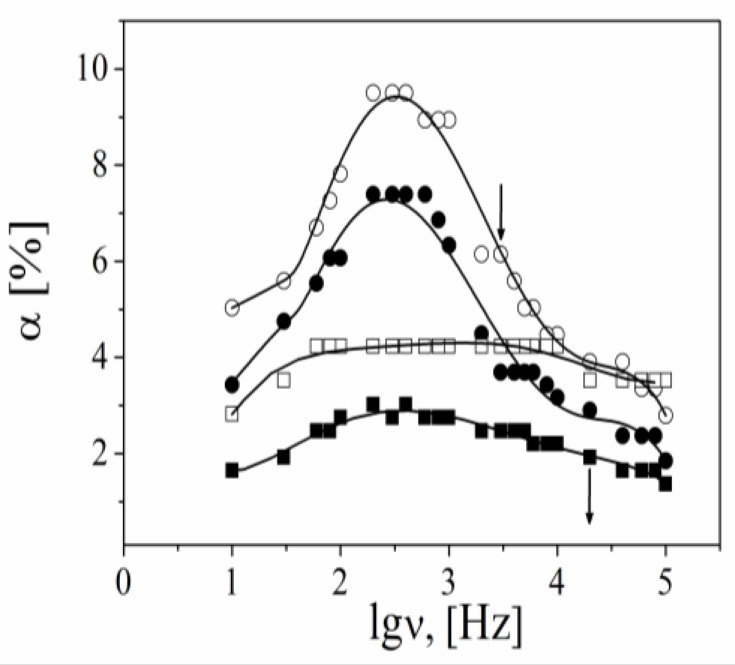
Dependence of the electro-optical effect, α, as a function of the frequency of the applied electric field from suspensions of particles with an adsorbed layer of ALG2 (●), ALG8 (○), CS2 (■), and CS8 (□). The electric field strength is 2.3 × 10^−4^ Vm^−1^. The arrows indicate the relaxation frequency of the effect.

**Figure 9:**
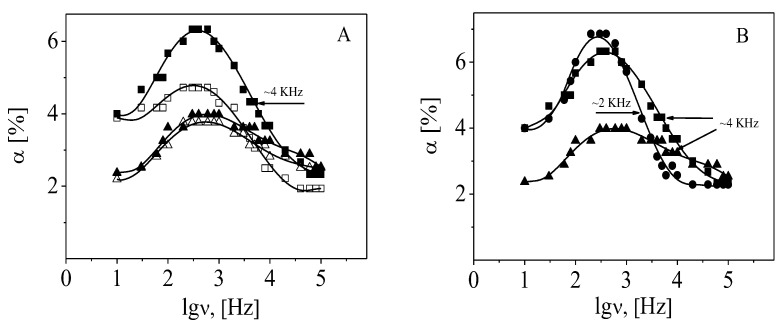
Dependence of the electro-optical effect, *α*, as a function of frequency of the applied electric field for (**A**) suspension of particles with last adsorbed layer CS1 (□), CS2 (■), CDs1 (△), and CDs2 (▲) and (**B**) suspension of particles with ALG2 (●), CS2 (■), and CDs2 (▲). The electric field strength is 2.3 × 10^−4^ V/m. The arrows indicate the relaxation frequency of the effect for particles.

Following the theory, the effect from the β-FeOOH particles results from the polarization of free Cl^−^ counterions along the particle axis, and the theoretical value (ν_cr_~30 kHz) is very close to the experimental one [35]. However, as can be seen in Figure 8 and Figure 9, the experimental value of the relaxation frequency of the effect is one order lower for the polymer-coated particles than for the bare particles (ν_cr_ ALG layer~2 kHz, ν_cr_ CS layer~5 kHz). 

One possible explanation for the decrease observed in ν_cr_ for the particles covered by a polymer might be the increase in the particle dimensions after each step. Hence, υ_cr_ will result from the polarization of free Na^+^ (for ALG) or Cl^−^ counterions (for CS) molecules along the particle axis [46]. According to Equation (6), a strong correlation between ν_cr_ and L_H_ can be expected, or υ_cr_ has to decrease with the number of steps (D_i_ is the translational diffusion coefficient of the condensed counterion and L is the polyion contour length) [47]:

ν_cr_ = 4D_i_/(πL^2^) (10)

However, the experimental results indicate that ν_cr_ does not depend on the number of adsorbed steps for each polymer and that there is dependence only on the type of polymer deposited in the outer layer. Furthermore, the decreasing L_H_ upon alginate adsorption does not correlate with the increase in ν_cr_ (or the opposite for the CS layers). 

The other explanation for the observed decrease in ν_cr_ is related to the influence of the charge density of the polymer in the last deposited layer. 

It is known that CS is a weak polyelectrolyte (the pKa value of glucosamine segments is 6.3–7.0) [48]; thus, it leads to a protonation under acidic conditions. At low pH, the intrinsic viscosity increases rapidly with decreasing ionic strength. Therefore, because of the strong electrostatic monomer–monomer repulsion, CS molecules adopt an extended and very flat conformation upon adsorption on an oppositely charged surface [49]. In the present experimental conditions, CS is regarded as an almost fully charged cationic polyelectrolyte whose charge density strongly depends on the degree of acetylation. As CS is dissolved in hydrochloric acid, Cl^−^ are the predominant counterions of the molecule. Similarly, the carboxyl groups of both mannuronic acid (pKa~3.38) and glucuronic acid (pKa~3.65) in the structure of ALG molecules can be dissociated by the addition of inorganic acids [21]. Therefore, the ALG has fully charged polyanionic chains. 

The classical counterion condensation theory describes the interactions of counterions with the highly charged polyion [30]. It predicts that some of the monovalent ions will condense into a volume tightly surrounding the chain if the average distance between two charges along the polymer chain, b, is smaller than the Bjerrum length, l_b_, (l_b_ = 0.714 nm in water at room temperature). The dimensionless linear charge density, related to the polyelectrolyte charge density, is defined as ξ = l_b_/b. 

The distance between the charges along the chitosan molecule is related to the DA by b = 0.515/(1 − DA), where 0.515 nm is the length per monosaccharide unit [50]. According to the theory, the fraction of free Cl^−^ counterions in a CS solution can be calculated by using the relation β=1/ξ, (at DA~15%, b = 0.64 nm, ξ = 1.11). Therefore, the presence of condensed counterions can be expected. 

The lengths of guluronic acid and mannuronic acid residues in the molecules of alginate are 0.435 nm and 0.517 nm, respectively. Therefore, one can conclude that in both cases counterion condensation also can be expected. Despite the polyelectrolyte nature of ALG, its molecules do not possess a defined monomer unit, and we can calculate the dimensionless linear charge density in the blocks (ξ is 1.64, 1.38, or 1.5 in G-block, M-block, or MG-block). The calculation of ξ for each homopolymeric domain does not violate the general assumption for the condensation theory, and the charge phenomena are similar to those of the typical polyelectrolytes [51,52]. Therefore, we confirm that the polarizability of CC is responsible for the different electro-optical behaviors of particles with adsorbed fully charged polyelectrolytes [53]. The relaxation frequency of the electro-optical effect can be estimated according to Equation (6).

The comparison between the experimental and the theoretical values of the frequency of relaxation is complicated because of the polydispersity of the CS sample used in this study. 

Moreover, because of the inhomogeneous structure of the ALG molecule, it is difficult to estimate the length and structure of the blocks. Therefore, the polyion contour length was calculated using Equation (6) from the experimental values of ν_cr_ (D_i_ (Na+) and is 9.8 × 10^−7^ cm^2^/s [54]). The estimated value (ca. 250 nm) was small compared to the length calculated from the molecular characteristics (ca. 1350 nm). Therefore, we assumed that the polarization of condensed Na^+^ counterions was on the small distance (for example G-, M- or MG-block) along the polyion contour. 

The presented results are in line with our previous studies and confirm the suggestions that the ν_cr_ of the effect depends on the polyelectrolyte charge density but does not depend on the number of deposition steps from the same polyelectrolyte and that the electrical properties of the polymer adsorbed in the last adsorption step define the electro-optical behavior of the film [32,33,34,47].

The registered effect decreased after the impregnation of the chitosan layer with Cdots (Figure 8). The effect of suspension with adsorbed CDs2 was higher when compared with that of CDs1 because of the higher amount of polysaccharide (or Cdots) on the increasing surface of the particles with the number of the deposition steps. According to the presented results, the values of ν_cr_ after Cdot adsorption were almost equal to those of the CS layer. Therefore, we suggest that the effect results predominantly from the polarization of condensed Cl^−^ counterions from the vicinity of the adsorbed CS molecules and assume that the participation of diffuse H^+^ counterions of Cdots is negligible.

### 3.4. Estimation of the Concentration of Cdots in the Film

**Figure 10:**
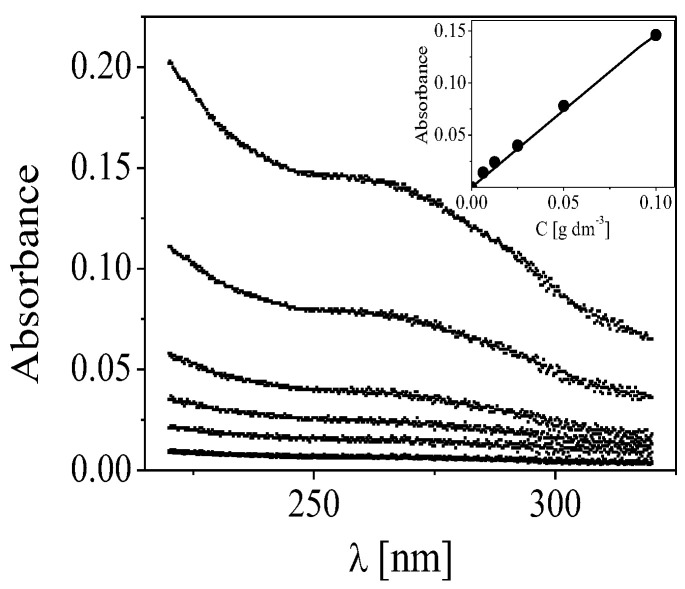
Absorbance from dispersion of Cdots at different concentrations. The curve with the lowest absorbance corresponds to the supernatant of the suspension of particles coated with polysaccharide film in the presence of CDs2. Inset: calibration curve of absorbance from dispersion of Cdots at different concentrations (0.62 × 10^−2^, 1.25 × 10^−2^, 2.50 × 10^−2^, 5 × 10^−2^, 10^−1^ mg/mL).

The loaded amount of Cdots was calculated from the difference between the concentrations of Cdots used in the adsorption (n × 0 µg/mL, n is the number of Cdot adsorption steps) added to the initial dispersion and those in the supernatant after centrifugation. For the estimation of the fraction of dots loaded into the film was used that the concentration of Cdots added to the dispersion for the formation of the two CDs layers was 20 µg/mL and for formation of eight CD layers was 80 µg/mL. The Cdots were detected at a wavelength of 278 nm, which corresponds to the maximum absorbance peak, and the concentration of free Cdots was calculated using an appropriate calibration curve (y = 1.478 x) (Figure 10).

The concentration of Cdots corresponds to 12.4 µg/mL (ca. 62% from the initial concentration, for Cdots2) and 56 µg/mL (ca. 70% from the initial concentration, for Cdots8). Therefore, the increment of the fraction of Cdots in the film is ca. 6.6 µg/mL per layer. The result indicates that a high amount of Cdots could be achieved by a control of the number of deposited CS/ALG bilayers on the particles. 

## 4. Conclusions

Negatively charged Cdots were loaded in a CS/ALG multilayer formed through sequential electrostatic adsorption on particles. The electrical properties of the film were studied using the electric light scattering method and electrokinetics. 

The registered electro-optical behavior for the suspension of polymer-coated particles indicates that the estimated values of the electro-optical effect (or electric polarizability) of the particles covered by the outer layer from ALG were significantly higher compared to the particles with the adsorbed CS layer due to the higher charge density of ALG. The presence of a low concentration of Cdots in the film resulted in the partial charge screening of the CS layer. 

It was confirmed that the polarizability of counterions with lower mobility was responsible for the obtained electro-optical results from the suspensions of particles with adsorbed fully charged polysaccharide chains and that the participation of the diffuse H^+^ counterions of the Cdots was negligible. 

The oscillation behavior in the evolution of the film thickness was interpreted through the participation of compensatory effects due to the additional adsorption/desorption of CS/ALG complexes from the film surface. 

The concentration of Cdots in the film was determined after adsorption, and the loaded amount was ca. 6.6 µg/mL per ALG/CS bilayer. 

Consequently, the working hypothesis was confirmed—the electrostatic interactions in the film were high enough to ensure the formation of stable polysaccharide/Cdots structures. Despite the registered oscillation of the dependence of electrical polarizability and thickness, both parameters slightly increased with the number of deposition steps. This can be taken as proof of a successful multilayer build-up process. 

## Data Availability

Not applicable.
